# The Sounds of Softness. Designing Sound for Human-Soft Robot Interaction

**DOI:** 10.3389/frobt.2021.674121

**Published:** 2021-10-12

**Authors:** Jonas Jørgensen, Mads Bering Christiansen

**Affiliations:** Center for Soft Robotics, SDU Biorobotics, University of Southern Denmark, Odense, Denmark

**Keywords:** soft robotics, human-robot interaction, sound design, social robotics, practice-based artistic research

## Abstract

In this article, we report on research and creative practice that explores the aesthetic interplay between movement and sound for soft robotics. Our inquiry seeks to interrogate what sound designs might be aesthetically engaging and appropriate for soft robotic movement in a social human-robot interaction setting. We present the design of a soft sound-producing robot, SONŌ, made of pliable and expandable silicone and three sound designs made for this robot. The article comprises an articulation of the underlying design process and results from two empirical interaction experiments (*N* = 66, *N* = 60) conducted to evaluate the sound designs. The sound designs did not have statistically significant effects on people’s perception of the social attributes of two different soft robots. Qualitative results, however, indicate that people’s interpretations of the sound designs depend on robot type.

## Introduction

Both in real life and in science fiction movies, there exist several examples of different nonverbal and nonlinguistic sounds that robots emit as they move about or manipulate objects. Often these sounds are of a mechanical character and result from e.g. the rotations of an electrical motor, the grinding of metal parts in a joint or in a linear actuator, or the hydraulic extension of a piston. Within the cultural imaginary, robotic sounds resulting from actuation and movement thus arguably comprise their own separate category with certain established expectations and conventions associated. But what happens if the functional rigid mechanical parts responsible for these emissions of sound are replaced by pliable and soft components?

In the past two decades, *soft robotics* has become a rapidly expanding research field with an increasing number of publications each year (Bao et al., 2018). Soft robotics research seeks to replace conventional components used for building robots with pliable and elastic ones, to gain functional advantages such as energy efficiency, increased maneuverability in unstructured environments, and increased safety through passive compliance for tasks that require close human-robot interaction (Abidi and Cianchetti, 2017; Luo et al., 2017; Santina et al., 2017; Wang et al., 2017).

At present, most soft robots are pneumatically actuated with electrical pumps or compressors, but actuators without mechanical sound based on e.g. dielectric elastomers, shape memory alloys and polymers, or biological cells are gradually becoming more common (El-Atab et al., 2020; Walker et al., 2020). Hence, in the future, soft robots may become practically devoid of sound. With which sounds should a soft robot’s movements and actions then be made audible to ensure safe, intuitive, and enjoyable interactions with humans?

In this article, we report on research and practice that explores the interplay between movement and sound in relation to how people experience a soft robot. More specifically, our inquiry seeks to interrogate what sound designs might be aesthetically engaging and appropriate for soft robotic movement within a social human-robot interaction setting. We present the design of a soft sound-producing robot made of pliable and expandable silicone and methods that we have used to design sound for soft robots anchored in practice-based artistic research. The article comprises an articulation of the underlying design process and two empirical experiments that examine what effect different sound designs have on people’s social perception of two different types of soft robots.

This article thus addresses the following three research questions:

• RQ1: What does a soft robot sound like and what is “soft” sound?
• RQ2: What effect does “soft” sound have on people’s social perception of a soft robot?
• RQ3: Are “soft” sounds a more appropriate match for a soft embodiment?

In relation to the wider theme of this special issue, the article contributes methodologically by illustrating and detailing how creative approaches and artistic methods can be integrated into human-robot interaction (HRI) research and contribute to articulating other questions and provide paths to novel insights. In addition, it presents a technical system designed to generate sounds to accompany soft robotic movement as a means of nonverbal signaling to human users. Finally, we report the results from a user study conducted to shed light on how sound affects people’s assessment of a soft robot’s sociality.

## Related Work

We position the work in the context of research on soft robotics, human interaction with soft robots, and sound design for robots.

Soft robots can be defined as systems that are capable of autonomous behavior and primarily composed of materials with elastic moduli in the range of that of soft biological materials (Rus and Tolley, 2015). Soft robots are claimed to offer inherently safer interactions with humans (Laschi et al., 2016), yet only a few publications have addressed how humans experience soft robots and how intuitive and engaging human interaction with them might be designed (Jørgensen, 2017a; Zheng, 2017; Boer and Bewley, 2018; Hu et al., 2018; Jørgensen, 2018; Shutterly et al., 2018; Zheng, 2018; Milthers et al., 2019; Jørgensen and Ploetz, 2020; Jørgensen et al., 2021; Zheng and Walker, 2019). Soft robotics technology has recently made its way into art, design, and architecture projects (Jørgensen, 2017b; Jørgensen, 2019). Yet adding sound to soft robots has not been explored within academic research and, to the best of our knowledge, only once within another creative practice project (Budak et al., 2016).

Sound has been argued to be a vital element of human communication and interaction, which should be supported in HRI. A number of HRI publications have called for more focus on sound, but robot sound design is still a nascent field of research. The addition of sound to robots has been argued to potentially improve human communication with robots and allow for more complex and meaningful interactions (Duffy, 2003; Cha et al., 2018; Jeong et al., 2017). Sound signals may also be more effective than visual cues for conveying emotional states in social robotics (Jee et al., 2009) and in HRI sound can be used to engage, inform, convey narratives, create affect, and generate attention (Schwenk and Arras, 2014). Research on robot sound design has taken many different forms including the voice-based teacher robot, *Silbot* (Jee et al., 2010), interactive sound generation with the humanoid *Robot Daryl* (Schwenk and Arras, 2014), Breazeal’s sociable infant robot *Kismet* with childlike sounds (Breazeal, 2002), as well as studies investigating people’s aural impressions of servo motors (Moore et al., 2017).

While many research efforts have centered on recreating human or animal sounds and human speech artificially (Duffy, 2003), recent research also exists that challenges this approach. It has been argued, for instance, that mimicking human or animal sounds could raise false expectations about a robot’s abilities (Schwenk and Arras, 2014).

Prior studies on *nonlinguistic utterances* (*NLUs*) as communicative and affective means of social robotics (Read and Belpaeme, 2010; Read and Belpaeme, 2012; Read and Belpaeme, 2013; Read and Belpaeme, 2014a; Read and Belpaeme, 2014b; Rosenthal-von der Pütten and Straßmann, 2018; Wolfe et al., 2020) have used highly varied sets of discrete machine-sounding audio cues, similar to the blips and bleeps of robots in sci-fi movies. Unlike these, the sound-producing system we use here was designed to generate a coherent soundscape to accompany and augment the robot’s movements and behaviors. Moreover, albeit using synthesizers for audio generation, our sound designs were purposely designed to embody both organic and machine-like qualities, and in these respects differ from research on NLUs (see *Design* detailing the design).

## Materials and Methods

### Methodology

**FIGURE 1:**
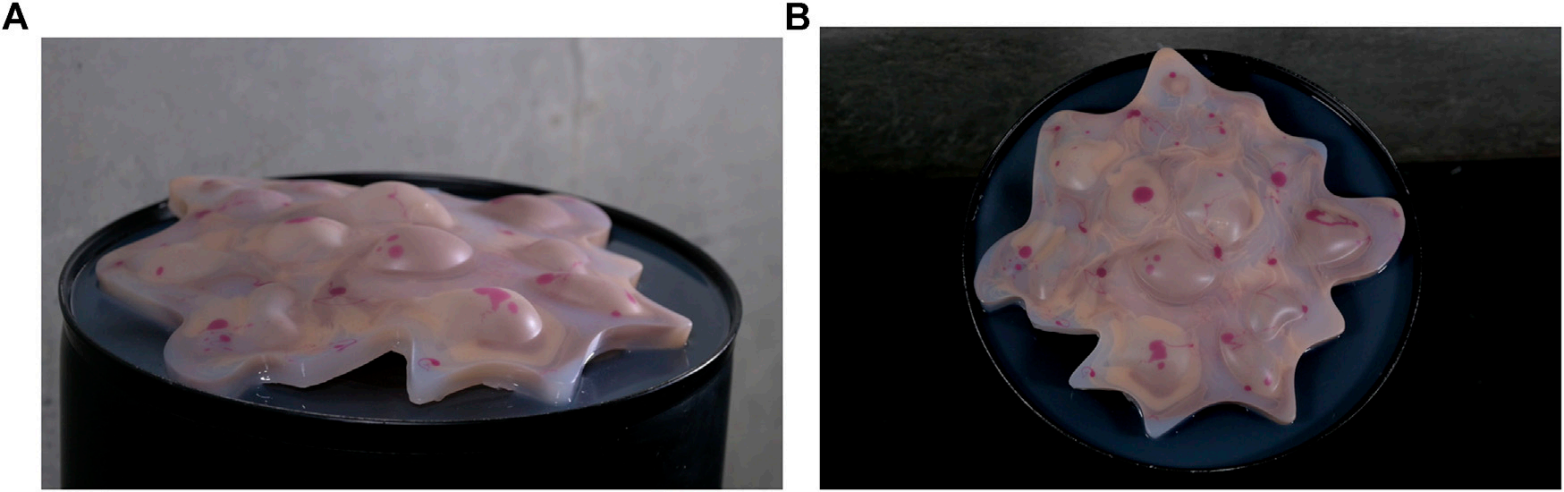
The SONŌ robot. Side view **(A)** and top view **(B)**.

We designed and fabricated a custom pneumatically actuated soft robot, *SONŌ* (Figure 1), and set up two interaction experiments that investigate how different sound designs influence people’s perception of a soft robot’s social attributes.

The design of the SONŌ robot and its sound is anchored in practice-based artistic research drawing on both authors’ practices within robotic art, electronic music, and sound design. Artistic research has, within the past two decades, been theorized as a specific mode of knowledge production. It can broadly be described as research in and through art practice that seeks to make present and communicate aesthetic experiences gained in creative practice and embodied in artistic products (Borgdorff, 2010). Artistic research diverges from artmaking in general, as it encompasses an ambition to contribute toward thinking and understanding and not just the development of an art practice in itself. It is linked to and engages with wider research communities, areas, or issues and hence by definition entails more than just the production of artworks. Methodologically, artistic research differs from traditional types of academic research in a number of respects. For instance, the requirement that a research study sets out with well-defined questions, topics, and problems, is at odds with the experimental character of art. Artistic research is instead undertaken on the basis of intuition, guesses, and hunches and is characterized by being open to serendipitous discoveries made along the way. Moreover, the exploration and navigating of unknown aesthetic and conceptual territories is facilitated by tacit understandings, accumulated experience, and artistic sensitivities rather than by pursuing answers to explicitly stated, rigorous, and unambiguous research questions *via* formalized methods. Hence, artistic research is discovery-led and not hypothesis-led in character (Borgdorff, 2010; Borgdorff, 2013).

We utilized practice-based artistic research methodology to address RQ1 (“What does a soft robot sound like and what is “soft” sound?”). We conducted an empirical user study, using established human-robot interaction methods and tools to evaluate the artistic outcomes in the context of HRI research and answer RQ2 (“What effect does “soft” sound have on people’s social perception of a soft robot?”) and RQ3 (“Are “soft” sounds a more appropriate match for a soft embodiment?”). The article thus extends prior work that has studied or evaluated robotic artworks and robot prototypes made by artists through empirical HRI experiments and prior work on leveraging the embodied meaning-making skills of artists to design robots (Demers, 2014; Vlachos et al., 2016, 2018; Levillain et al., 2017; LaViers et al., 2018; Cuan et al., 2018a; Cuan et al., 2018b; Gemeinboeck and Saunders, 2018; Gemeinboeck and Saunders, 2019; Herath et al., 2020).

### Design

The practice-led research started out from the speculative question “What does a soft robot sound like?”. Our intention was to experiment with how incorporating sound into a soft robot could add to its qualities and to explore how sound might support the inherent aesthetic qualities of soft robotics technology [early work has previously been reported in a Late-Breaking Report and a video (Bering Christiansen and Jørgensen, 2020a; Bering Christiansen and Jørgensen, 2020b)]. As research shows people to have a better impression and understanding of products and designs where two or more sensuous modalities are coupled (Langeveld et al., 2013), we chose to focus on how sound might augment soft robotic movement.

#### Design and Fabrication of the SONˉO Robot

**FIGURE 2:**
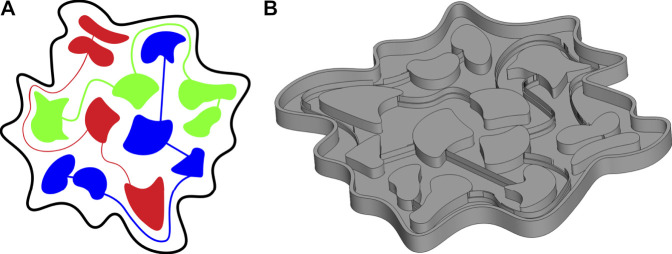
SONŌ air chamber overview **(A)** and CAD rendering of the mold **(B)**.

In our design of the robot morphology we aimed for a design that would be perceived as organic yet unfamiliar. We chose a non-anthropomorphic and non-zoomorphic form and used abstract rounded shapes and a main color similar to Caucasian skin with reddish colorations to give the robot organic connotations. We opted for a simple design with only three independent pneumatic channels that each connect 4 chambers that can expand upon inflation and are located across the morphology (Figure 2). We deemed this design to provide sufficient possibilities for variation in realizable expressive movement.

The soft morphology was cast from Ecoflex 00-30 silicone colored with Silc-Pig pigments in a 3D printed mold (Figure 2B), using the following fabrication procedure. Three different containers with liquid silicone were mixed and degassed in a vacuum chamber. The first contained a light Caucasian skin tone-like pigment, the second a delicate pink pigment, and the third uncolored semitransparent silicone. The three liquid silicones were mixed directly inside the mold. A coloring with similarities to the faux marble paint effect was created by switching between the three liquid silicones when pouring them into the mold. Finally, smaller dots of deep red/purple pigmented silicone were dripped into the uncured silicone surface from a 20 cm distance with a small brush. The cured top part was removed from the mold and cast onto a strain limiting bottom piece consisting of precured silicone-coated nonwoven mesh (Vlieseline S13). Finally, three transparent supply tubes in PVC with a length of 90 cm each and 1.5 mm/3 mm ID/OD were inserted into each pneumatic channel of the soft morphology from below and the robot was coated with talc powder to prevent lint and dust from adhering to it.

#### System Overview and Technical Setup

**FIGURE 3:**
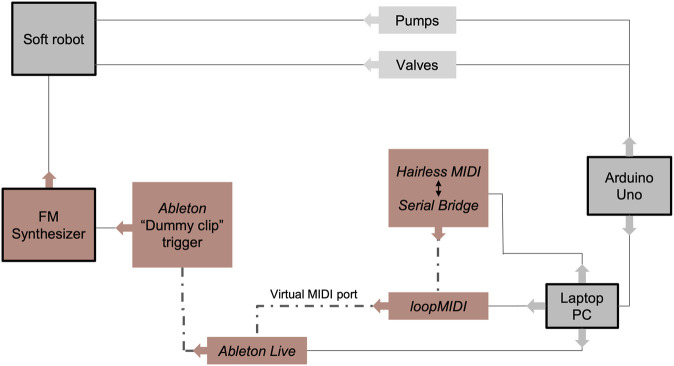
Diagram of the technical system. Gray boxes indicate physical elements and red boxes software applications and protocols.

Figure 3 shows an overview of the system. The three physical main components are the soft robot morphology, an Arduino UNO microcontroller, and a laptop PC with a connected active speaker. The Arduino is equipped with a custom-made motor shield that drives three low noise pumps (MITSUMI R-14 A213) and three solenoid valves (Uxcell Fa0520D 6V NC) to control the soft robot’s inflation and release of air.

The SONŌ robot does not currently possess any sensors for feedback control. It uses an open-loop control and switches between preprogrammed movement sequence that are executed by activating the three pumps and three valves with manually programmed time delays (Arduino code available as Supplementary Material). This creates bulges on the top part where the compartments are found. Expressive movement primitives of the morphology were discovered empirically through aesthetic experimentation with the robot and were combined to form programmed movement sequences. In parts of the movement sequences only one pneumatic channel is actuated, whereas in others two or all three channels inflate or deflate simultaneously. The movements performed by this and the other robot used for the interaction experiments are demonstrated in the accompanying video (Youtube link: https://youtu.be/vKHTJe8t-R0).

#### Frequency Modulation Synthesis

We used *frequency modulation* (*FM*) synthesis to design sound for the robot due to this technique’s customizability and malleability and because it is argued to recreate natural sounds better than other forms of analogue synthesis (Jenkins, 2007). FM synthesis is based on pitch modulation of one or more oscillators (Jenkins, 2007). An FM synthesizer consists of *operators*, a term used to describe individual oscillators with separate amplitude envelopes. The amplitude of one or more *modulator operators* affects the frequency of the *carrier operator* through an algorithm, i.e. the configuration of how multiple operators interact. Depending on the algorithm, an operator can modulate other operators, be modulated by other operators or both, which has a substantial effect on the synthesizer’s sound and timbral qualities with no use of filters. With FM synthesis it is possible to generate sound designs with rich complex harmonics that are impossible to create with other synthesis techniques (Chowning and Bristow, 1987).

#### Audio Generation

Audio to accompany the robot’s movements is generated in real time by the software synthesizer *Operator* running within the *digital audio workstation (DAW)*, *Ableton Live*, on a laptop computer. The microcontroller sends MIDI signals to the DAW by utilizing a serial connection-to-MIDI-bridge, *Hairless MIDI*, and a virtual loopback MIDI-port, *LoopMIDI* (detailed setup guide included as Supplementary Material). The MIDI signals are sent via serial connection over USB when a pump or valve is switched on which triggers a note on the FM synthesizer. When an air chamber inflates, the frequency of the carrier operator and modulator operator(s) increases, and it decreases when an air chamber deflates. In the current setup, the robot switches between preprogrammed movement sequences, and accompanying MIDI messages sent from the microcontroller trigger “MIDI dummy clips.” Dummy clips are silent MIDI clips within Ableton Live that contain an automation for modulating certain parameters of one or more devices—in this case the Operator FM synthesizer’s oscillator and filter cutoff parameters. Different dummy clips have been created to contain actions that fit both inflation and deflation of the soft robot: if an air chamber inflates, a dummy clip containing inclining oscillator curve manipulation is triggered, and if an air chamber deflates, another clip containing declining oscillator curve manipulation will play. Multiple dummy clips to each sound design have been added to the DAW to allow for a less static sound image. The dummy clips have different lengths and are triggered selectively in the microcontroller control code so that they match the time a specific inflation or deflation takes, i.e. the sound does not stop abruptly.

Three sound designs were made as individual *patches*, preconfigured combinations of oscillators, filters, and envelope settings (Roland, n.d.), for the FM synthesizer. Technical details on the three patches and Ableton patch files are included as Supplementary Materials.

#### First Sound Design: “Movies”

A sound’s identity—its spectro-temporal characteristics such as pitch, timbre, duration, and level—and the location of its source allows people and animals to extract relevant information from audio (Carlile, 2011). Auditory perception relies on information derived from these features that is recombined in the brain into useful and decodable signals (Carlile, 2011). Every sound and acoustic event can be understood as a decodable sign carrier that communicates information (Jekosch, 2005). For living creatures, a distinction can be made between *internal* and *external auditory cues* (Cha et al., 2018). Internal auditory cues are sounds entirely generated by the creature’s own body such as breathing, snoring, or sighing, while external auditory cues are produced by its physical interaction with the environment. Echoing this distinction, commercial sound designers differentiate between *consequential sounds* and *intentional sounds* (Langeveld et al., 2013). Consequential sounds occur due to the mechanical functioning of a product’s parts, intentional sounds are auditory instances meant to be triggered when products interact with their surroundings (Langeveld et al., 2013). Consequential sounds, e.g. actuation sound coming from electrical motors, are often regarded as noisy and are restricted by the physical design and properties of the product. Intentional sounds, on the contrary, are deliberate and designed.

As we did not want our initial sound design to directly mimic animal and human sounds, we started out by studying sounds made by imaginary soft characters portrayed in movies. We sought to familiarize ourselves with this existing pop-cultural frame of reference, to gain an understanding of what soft entities have been imagined to sound like and to attain insight into how their sound designs have been generated. We chose this approach as we reasoned that aligning our “soft” sound design with the formal traits of this existing repertoire of “soft” sounds could yield recognizability and make the listener associate the sound design with (fictional) soft beings. Initially, movies that contain characters with soft bodies and/or morphing/deformable soft tissue were identified by searching the internet and going through user lists on the online movie database *IMDb* (https://www.imdb.com/). Summaries and trailers for relevant movies were screened and based on this process we identified 10 movies wherein sound was a prominent feature of a soft imaginary character [*Alien* (Scott, 1979), *Alien vs. Predator* (Anderson, 2004), *Flubber* (Mayfield, 1997), *Night of the Creeps* (Dekker, 1986), *Slither* (Gunn, 2006), *Spiderman 3* (2007), *Terminator 2* (Cameron, 1991), *The Blob* (Yeaworth and Doughten, 1958), *The Thing* (Carpenter, 1982), and *Venom* (Fleischer, 2018)]. Both authors studied clips of each of these movie characters and wrote notes on what characterized their sound and how it changes upon interaction, differentiating between internal and external auditory cues. We discussed these notes and mapped shared defining features of the characters’ sounds that could be considered vectors spanning the sound design space of the soft movie characters. We made the following general observations:

• Sound is dynamic (there are often rapid changes in the sound)
• Two strategies for generating sound are prevalent: 1. Recorded sounds from animals are layered, 2. Layered sounds from synthesizers are used
• Sounds are often manipulated by raising or lowering pitch or using filters. This creates “wet” or “slippery” sounds, which change in accordance with the character’s movements
• Internal auditory cues convey the character’s state of mind and mood. External auditory cues provide information concerning the character’s movements and physical interaction with the environment

Utilizing the above observations as design guidelines, we created the SONŌ robot’s first sound design patch named “Movies.” The patch uses two square wave modulator operators, whose frequencies are modulated in opposite directions through a dynamic lowpass filter when movement occurs. The FM synthesizer is routed through a virtual tape echo delay with a short delay time and a high feedback percentage, which results in frequency fluctuations and overall frequency manipulation.

#### Second Sound Design: “White Noise”

For the second sound design, we wanted to design a “soft” sound using a different approach. We started by discussing what we understand a “soft” sound to be, in order to articulate our accumulated tacit understandings gained through experience in creative sound practice, and came to agree on some general characteristics (long envelope, timbre/spectrum not high-pitch, gradual/slow changes, lowpass filter smoothing). From this starting point, we further researched how “soft” sound is described in the literature.

In relation to sound, there are different ways in which “soft” can be understood. Dictionaries describe “soft” sound as “quiet in pitch or volume” (Merriam Webster, 2021), “gentle” and “not forceful” (Cambridge Dictionary, 2021), something “not harsh,” or “low and pleasing” (Collins Dictionary, 2021). Within psychoacoustics, loudness is understood as an attribute of auditory sensation ranging on a scale from “soft,” which describes low amplitude sounds, to “loud,” which describes high amplitude sounds (Lentz, 2020; Fastl and Zwicker, 2007). In relation to pitch and timbre, a “soft” sound is usually low-frequency (Cook, 1999) and has less brightness than a loud sound (Cook, 2011). However, simple tones with no timbral harshness and a lack of power in the low-frequency domain, such as sine tones, have equally been described as “soft” (Howard and Angus, 2009; Seidenburg et al., 2019). The word “piano,” which translates as “soft,” is also used within music theory to describe a decrease in a musical score’s intensity achieved by playing an instrument more gently, whereby not only the sound’s amplitude is changed, but also its timbral qualities (Cook, 1999).

As the above usages of the word “soft” illustrate, different meanings persist that each point to different physical characteristics of soft sound. In our design of the second “soft” sound we chose to disregard soft as the opposite of loud, and instead focus on soft sound as the opposite of hard or harsh sound.

The patch for the second “soft” sound, named “White Noise,” is based on a white noise signal. It produces a fizzing high-pitch sound with a dynamic filter cutoff. This gives the patch similarities to natural sounds such as wind or ocean waves. Based on the movements of the soft robot, the filter cutoff frequency, filter envelope percentage, and filter end position percentage are modulated in the sound design.

#### Third Sound Design: “Glass Attack”

As the third sound, we wanted to make a “hard” sound to contrast and compare the two “soft” sounds against. We came up with the idea to construct a sound design which sounds similar to a sound that can be produced by a hard object.

The third patch is a midtone sine wave with a relatively short amplitude attack time that includes many high-frequency harmonics, which contribute to a bell-like or glass-like sound, hence we gave it the name “Glass Attack.” It sounds somewhat similar to the sound emitted by a drinking glass when the glass is brought to resonate by gently rubbing a wet finger along the rim of the glass. When the robot moves, the synthesizer uses gliding pitch manipulation to indicate inflation and deflation. This produces a sound with similarities to the resonance or impact sounds of objects made from glass or metal, with a gliding pitch manipulation added to prevent the sound from becoming static.

### User Studies

**FIGURE 4:**
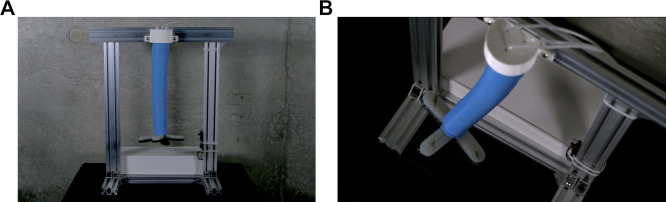
The tentacle robot. Front view **(A)** and top view **(B)**.

We conducted two interaction experiments to test the impact of the sound designs on people’s social perception of a soft robot. In the first (Experiment 1) the three sound designs were tested on the SONŌ robot, and in the second (Experiment 2) they were tested on another soft robot (Figure 4). This second robot is a pneumatically actuated soft silicone tentacle hanging from an aluminum frame, which was used in another study (Jørgensen et al., 2021). A four fingered soft robotic pneunets gripper cast in Ecoflex 00-30 was added to the tip of this tentacle (Finio, 2013). In the following, we will refer to this robot as the *Tentacle* robot.

We chose to test the sound designs on two different soft robot types to gain insight into whether the sound designs had similar effects, when used on soft robots in general, or if there were differences related to the type of robot using them. Both experiments had three conditions corresponding to the three sound designs, and in each experiment each robot performed the same preprogrammed movement sequence in every experiment condition (only the sound differed). We used a between-subjects design to gauge people’s first impressions of the robots and avoid bias due to carry-over effects. The experiments took place over 4 days at the University of Southern Denmark (Odense) in a classroom in the main university building.

### Participants

Participants were a convenience sample of people present, of whom all but one participant turned out to be university students. Demographic information for each condition is given in Table 2 and Table 3 under *4 Results*. Participants did not receive any compensation for their participation. Experiment 1 had a total of 66 participants and Experiment 2 had 60 participants.

### Data Collection

We used the Robotic Social Attributes Scale (RoSAS) to measure people’s impressions of the robot’s sociality and added additional questions to obtain information about their perception of the robot’s sound. The RoSAS scale is a validated tool that can be used to measure people’s impressions of a robot’s sociality (Carpinella et al., 2017). The scale measures three main constructs, with 6 subitems each: *Competence* (*Reliable, Competent, Knowledgeable, Interactive, Responsive, Capable*), *Warmth* (*Organic, Sociable, Emotional, Compassionate, Happy, Feeling*), and *Discomfort* (*Awkward, Scary, Strange, Awful, Dangerous, Aggressive*). Participants were asked: “Using the scale provided, how closely are the words below associated with the robot you have just experienced?”. Ratings were given on a 7-point scale (1—not at all, 7—very much so). We aimed to have at least 20 participants per condition as other studies using the RoSAS scale with a similar number of conditions have found this to give sufficient statistical power (Pan et al., 2018).

Before filling out the RoSAS scale, participants were asked to “Write the first three words that come to mind to describe the robot that you have just experienced,” following the method proposed by Damholdt et al. (2019). Another scale question was added after the RoSAS scale: “Using the scale provided, please indicate to which extent you agree with the following statement about the robot: ‘The robot has a sound that is appropriate for it’” (1-Strongly agree, 7-Strongly disagree). This question was followed by an open question asking people to elaborate on their choice of answer.

As the experiments took place on the campus of a Danish university, we translated the questionnaire into a Danish version. We pretested the Danish questionnaire with five participants who experienced a video equivalent of condition 1 of Experiment 1. We changed two translated words (“kapabel” to “duelig,” “responsiv” to “reaktionsdygtig”) that participants expressed difficulty in understanding.

### Procedure

The procedures for Experiment 1 and Experiment 2 were identical, only difference being the robot used and the pre-experiment briefing given to participants.

We asked people if they would like to participate in a research study on human-robot interaction, and upon acceptance they were accompanied to the classroom where the experiment would take place. Participants received information about the project and the experiment verbally and were given an information sheet and provided opportunity to ask questions. We did not specify to participants that the study’s focus was on sound, to avoid a bias in the RoSAS ratings of the robots, as the RoSAS scale is designed to assess the overall sociality of a robot and not a single aspect of it such as its sound. Withholding this information was approved by the university’s ethics committee, on the condition that it be provided in the debriefing. Written informed consent was obtained from participants, who were all above the Danish legal age of 18, both for participation in the experiment and for the collection of personal data.

**FIGURE 5:**
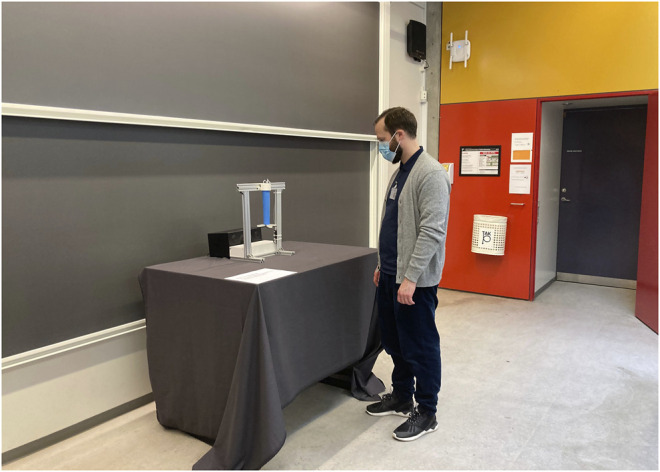
Photo of the experiment settings.

In the experiments, one of the robots was placed on a table covered in dark gray cloth (Figure 5). In Experiment 1 that used the SONŌ robot, the electro-pneumatic actuation and control system was hidden underneath the table inside a small enclosure made from mattress foam (to dampen mechanical sound), and only the soft morphology was clearly visible on the table. The Tentacle robot instead had the actuation and control system hidden inside an integrated white acrylic enclosure (Figure 4). For sound, we used a portable active speaker, which was placed under the table for the SONŌ robot and on the table behind the Tentacle robot (Figure 5). We adjusted the volume of the speaker to compensate for the dampening of the sound when placed under the table, so that the loudness of each sound design was experienced as approximately the same in the two experiments. We also made adjustments to ensure that the three sound designs were experienced as being of approximately the same loudness, with sound levels as given in Table 1. As can be seen from Table 1, the mechanical sounds produced by the robots themselves were lower than the sound designs playing on the loudspeaker and we estimate that they were barely noticeable during the experiments.

**TABLE 1:** Average and maximum sound levels (in decibel) for the sounds occurring in the two experiments. The sound levels of the sound designs have been measured without the robot moving. The mechanical sound of the robots themselves has been measured without any sound playing on the loudspeaker. The measurements were done with the robots and the loudspeaker positioned on the table and cloth as described above. A sound meter was positioned at a fixed distance of 1 m to the robot corresponding to the approximate distance and height at which the sound would be heard by a participant.

	Avg	Max
**1-SONŌ-White** (only sound design)	66.4	77.6
**2-SONŌ-Glass** (only sound design)	57.0	68.6
**3-SONŌ-Movies** (only sound design)	60.1	72.9
**4-Tent.-White** (only sound design)	63.0	71.2
**5-Tent.-Glass** (only sound design)	59.3	73.5
**6-Tent.-Movies** (only sound design)	58.5	68.2
**SONO robot** (no sound design)	43.7	56.9
**Tentacle robot** (no sound design)	38.7	45.1

We instructed participants that we would show them a robot, and that they should observe it and let us know when they felt ready to answer some questions about it. Participants experienced the robot individually or in groups of up to five persons for 2–6 min. Groups were formed as we recruited passersby on a hallway, where people were often walking in groups. We allowed a group of people to enter the premises together when several volunteered to participate at the same time. Owing to the Covid-19 pandemic we could not allow physical interaction with the two robots, hence the robots performed preprogrammed movements accompanied by sound and did not respond to participants. We encourage the reader to consult the accompanying video to get an impression of the robots and the three sound designs (Youtube link: https://youtu.be/vKHTJe8t-R0). For the SONŌ robot, participants were additionally asked to imagine that the robot was communicating and expressing itself through its movements and sound. We did this to frame this robot as a social robot. For the Tentacle robot, we instead briefed people to imagine that they were to solve a practical task, such as packing or moving small goods or products, together with this robot. We also explained that the attached soft gripper could grasp and pick up objects. We did this to frame the Tentacle robot as a soft collaborative robot (cobot).

Following exposure, participants filled out the questionnaire and provided selected demographic data (age, gender, level of familiarity with robots, prior human-robot interaction experience, field of study if a student at the university). Participants could choose freely between the Danish and the English version of the questionnaire, with 110 choosing the Danish version and 16 the English version. Finally, participants received a debriefing and were provided the opportunity to ask questions.

### Hypotheses

We hypothesized that a sound design with “soft” qualities would:

• Result in higher *warmth* and *competence* ratings and lower *discomfort* ratings, than one without these qualities.
• Be deemed more appropriate for a soft robot.
• Elicit word associations with a higher rate of positive sentiments.

## Results

### Robotic Social Attributes Scale Ratings and Appropriateness

The internal consistency of the RoSAS data was confirmed by two internal reliability tests performed on the complete data set. We calculated Cronbach’s alpha, a commonly used measure of the internal consistency reliability among a group of items that form a scale. We additionally calculated the mean inter-item correlation, a more appropriate measure of internal consistency for scales with less than ten items (Briggs and Cheek, 1986). Cronbach’s alpha values of 0.75 for *competence*, 0.73 for *warmth*, and 0.75 for *discomfort* were obtained, which are above the standard 0.70 threshold, indicating an acceptable internal consistency. The mean inter-item correlations were 0.33 for *competence*, 0.33 for *warmth*, and 0.34 for *discomfort* and fall within the optimal range of 0.2–0.4.

We used one-way between-groups analysis of variance (ANOVA), χ2 test for independence, and Welch test, as appropriate, to assess whether age, gender, and mean values of each the three RoSAS scale main constructs differed for the three conditions in each experiment. The same methods were used to determine if there were differences in how appropriate the sound designs were rated to be for the two robots. The results for the two experiments are given in Table 2 and Table 3.

**TABLE 2:** Results and demographic data from Experiment 1. Groups under “Faculty” indicate under which faculty the participant studies if a student at the university (HUM = humanities, NAT = natural sciences, SOC = business and social sciences, HEA = health sciences, TEC = technical sciences).

	1-SONŌ-White (*N* = 20)	2-SONŌ-Glass (*N* = 21)	3-SONŌ-Movies (*N* = 25)	N	P-value(ANOVA/χ2/Welch)
**Competence**	M: 3.48 SD:1.01	M: 3.00 SD:1.05	M: 3.47 SD:1.11	66	0.25
Reliable	M: 3.65 SD:1.50	M: 3.33 SD:1.32	M: 2.76 SD:1.13	66	0.08
Competent	M: 3.40 SD:1.47	M: 2.95 SD:1.66	M: 3.36 SD:1.35	66	0.56
Knowledgeable	M: 2.65 SD:1.50	M: 2.76 SD:1.76	M: 3.32 SD:1.55	66	0.32
Interactive	M: 3.50 SD:1.96	M: 2.95 SD:1.80	M: 3.56 SD:1.83	66	0.50
Responsive	M: 3.75 SD:1.68	M: 2.86 SD:1.68	M: 4.28 SD:1.75	66	0.02
Capable	M: 3.90 SD:1.52	M: 3.14 SD:1.28	M: 3.56 SD:1.45	66	0.24
**Warmth**	M: 3.19 SD:0.99	M: 3.14 SD:1.38	M: 3.49 SD:1.13	66	0.56
Organic	M: 5.35 SD:1.50	M: 4.67 SD:2.22	M: 4.36 SD:2.02	66	0.24
Sociable	M: 2.35 SD:1.39	M: 2.29 SD:1.38	M: 2.92 SD:1.55	66	0.27
Emotional	M: 2.75 SD:1.52	M: 3.48 SD:1.86	M: 3.44 SD:1.92	66	0.34
Compassionate	M: 2.55 SD:1.32	M: 2.52 SD:1.72	M: 2.76 SD:1.39	66	0.84
Happy	M: 2.85 SD:1.42	M: 2.43 SD:1.75	M: 3.12 SD:1.62	66	0.35
Feeling	M: 3.30 SD:1.98	M: 3.48 SD:2.09	M: 4.32 SD:1.75	66	0.17
**Discomfort**	M: 3.15 SD:0.98	M: 3.19 SD:1.08	M: 3.65 SD:1.14	66	0.22
Awkward	M: 3.75 SD:1.34	M: 3.29 SD:2.00	M: 3.48 SD:1.36	66	0.64
Scary	M: 3.30 SD:1.92	M: 3.43 SD:1.91	M: 3.64 SD:2.00	66	0.84
strange	M: 6.00 SD:1.56	M: 5.95 SD:1.16	M: 6.00 SD:1.35	66	0.99
Awful	M: 2.10 SD:1.65	M: 2.48 SD:1.60	M: 2.84 SD:1.60	66	0.32
Dangerous	M: 1.70 SD:1.41	M: 2.19 SD:1.44	M: 2.36 SD:1.60	66	0.33
Aggressive	M: 2.05 SD:1.43	M: 1.81 SD:1.25	M: 3.60 SD:1.68	66	0.00
**Appropriateness of sound**	M: 4.25 SD:1.71	M: 3.95 SD:1.63	M: 4.68 SD:1.63	66	0.33
Age	M: 23.5 SD:5.23	M: 23.9 SD:2.90	M: 22.1 SD:1.49	66	0.04
Gender (female/male)	(10/10)	(6/15)	(11/14)	66	0.35
Familiarity w. robots	M: 3.35 SD:1.73	M: 3.33 SD:1.91	M: 3.04 SD:1.97	66	0.82
Faculty (HUM/NAT/SOC/HEA/TEC)	(8/2/0/8/5)	(1/4/2/2/11)	(6/3/4/8/4)	66	—

**TABLE 3:** Results and demographic data from Experiment 2

	4-Tent.-White (*N* = 20)	5-Tent.-Glass (*N* = 20)	6-Tent.-Movies (*N* = 20)	N	P-value(ANOVA/χ2/Welch)
**Competence**	M: 3.48 SD:1.02	M: 3.69 SD:1.07	M: 3.23 SD:0.84	60	0.34
Reliable	M: 3.50 SD:1.40	M: 4.21 SD:1.65	M: 3.05 SD:1.32	59	0.05
Competent	M: 3.55 SD:1.43	M: 4.05 SD:1.50	M: 3.45 SD:1.47	60	0.39
Knowledgeable	M: 2.60 SD:1.27	M: 3.00 SD:1.26	M: 2.60 SD:1.43	60	0.55
Interactive	M: 3.85 SD:1.60	M: 3.75 SD:2.10	M: 3.10 SD:1.29	60	0.32
Responsive	M: 3.20 SD:1.24	M: 3.45 SD:1.43	M: 3.40 SD:1.67	60	0.85
Capable	M: 4.20 SD:1.44	M: 3.90 SD:1.48	M: 3.80 SD:1.58	60	0.68
**Warmth**	M: 2.78 SD:1.01	M: 2.28 SD:0.68	M: 2.58 SD:1.06	60	0.23
Organic	M: 3.90 SD:2.02	M: 3.35 SD:1.95	M: 3.15 SD:1.84	60	0.45
Sociable	M: 2.50 SD:1.54	M: 1.68 SD:0.95	M: 2.25 SD:1.86	59	0.23
Emotional	M: 2.05 SD:1.32	M: 1.55 SD:1.00	M: 2.05 SD:1.47	60	0.37
Compassionate	M: 2.20 SD:1.51	M: 1.50 SD:1.00	M: 1.85 SD:0.99	60	0.22
Happy	M: 3.20 SD:1.58	M: 2.40 SD:1.73	M: 2.95 SD:2.14	60	0.37
Feeling	M: 2.85 SD:1.46	M: 3.25 SD:1.83	M: 3.25 SD:1.71	60	0.69
**Discomfort**	M: 2.61 SD:1.18	M: 2.73 SD:1.07	M: 3.12 SD:1.11	60	0.33
Awkward	M: 3.30 SD:1.78	M: 3.85 SD:2.23	M: 4.80 SD:2.02	60	0.07
Scary	M: 2.80 SD:1.96	M: 2.20 SD:1.85	M: 2.50 SD:1.76	60	0.60
strange	M: 4.15 SD:1.95	M: 4.80 SD:1.99	M: 5.00 SD:2.10	60	0.39
Awful	M: 2.10 SD:1.45	M: 2.35 SD:1.69	M: 2.80 SD:1.64	60	0.38
Dangerous	M: 1.75 SD:1.12	M: 1.50 SD:0.83	M: 1.70 SD:1.34	60	0.76
Aggressive	M: 1.55 SD:1.00	M: 1.70 SD:0.33	M: 1.90 SD:1.21	60	0.68
**Appropriateness of sound**	M: 4.30 SD:1.92	M: 4.58 SD:1.81	M: 3.75 SD:2.12	59	0.41
Age	M: 22.6 SD:1.76	M: 23.0 SD:2.20	M: 25.0 SD:4.95	59	0.14
Gender (female/male)	(5/15)	(8/11)	(8/12)	59	0.47
Familiarity w. robots	M: 4.35 SD:2.16	M: 3.00 SD:1.53	M: 2.55 SD:1.47	59	0.01
Faculty (HUM/NAT/SOC/HEA/TEC)	(0/2/0/6/12)	(2/3/10/0/4)	(2/3/3/0/12)	59	—

We found that with respect to familiarity with robots, participants in condition 4 differed significantly from those in conditions 5 and 6 (*p* = 0.050 and *p* = 0.005 respectively). As the assumption of homogeneity was violated when comparing mean age between conditions for both experiments, we used the Welch test for this instead of ANOVA.

We found no statistically significant differences in *competence*, *warmth*, and *discomfort* ratings between the different sound design conditions in either Experiment 1 or Experiment 2.

In secondary exploratory analyses, we compared ratings for each of the RoSAS subitems between the three sound design conditions within each experiment. For experiment 1 we found a statistically significant difference (*p* = 0.023) for *responsive* between condition 2 (*M* = 2.86) and condition 3 (*M* = 4.28). A statistically significant difference (*p* = 0.000) for *aggressive* between condition 3 (*M* = 3.60) and both condition 1 (*M* = 2.05) and condition 2 (*M* = 1.81) was also found. For experiment 2 we found borderline statistically significant differences for *reliable* (*p* = 0.052) between condition 5 (*M* = 4.21) and condition 6 (*M* = 3.05) and for *awkward* (*p* = 0.067) between condition 4 (*M* = 3.30) and condition 6 (*M* = 4.80). We also compared how appropriate each of the three sound designs were rated to be with the SONŌ robot and the tentacle robot respectively, using T tests and data from both the experiments. We found no significant differences (*p* > 0.05) despite differing mean values (Table 2 and Table 3).

### Sentiment Analysis of Descriptive Words

Following the method described in (Damholdt et al., 2019), we used logistic regression to determine if the words used to describe the robots had different distributions of sentiment in the three sound conditions for each robot. We used this method of analysis to determine if the sound design condition could predict the likelihood that respondents would report a word with a positive sentiment.

**FIGURE 6:**
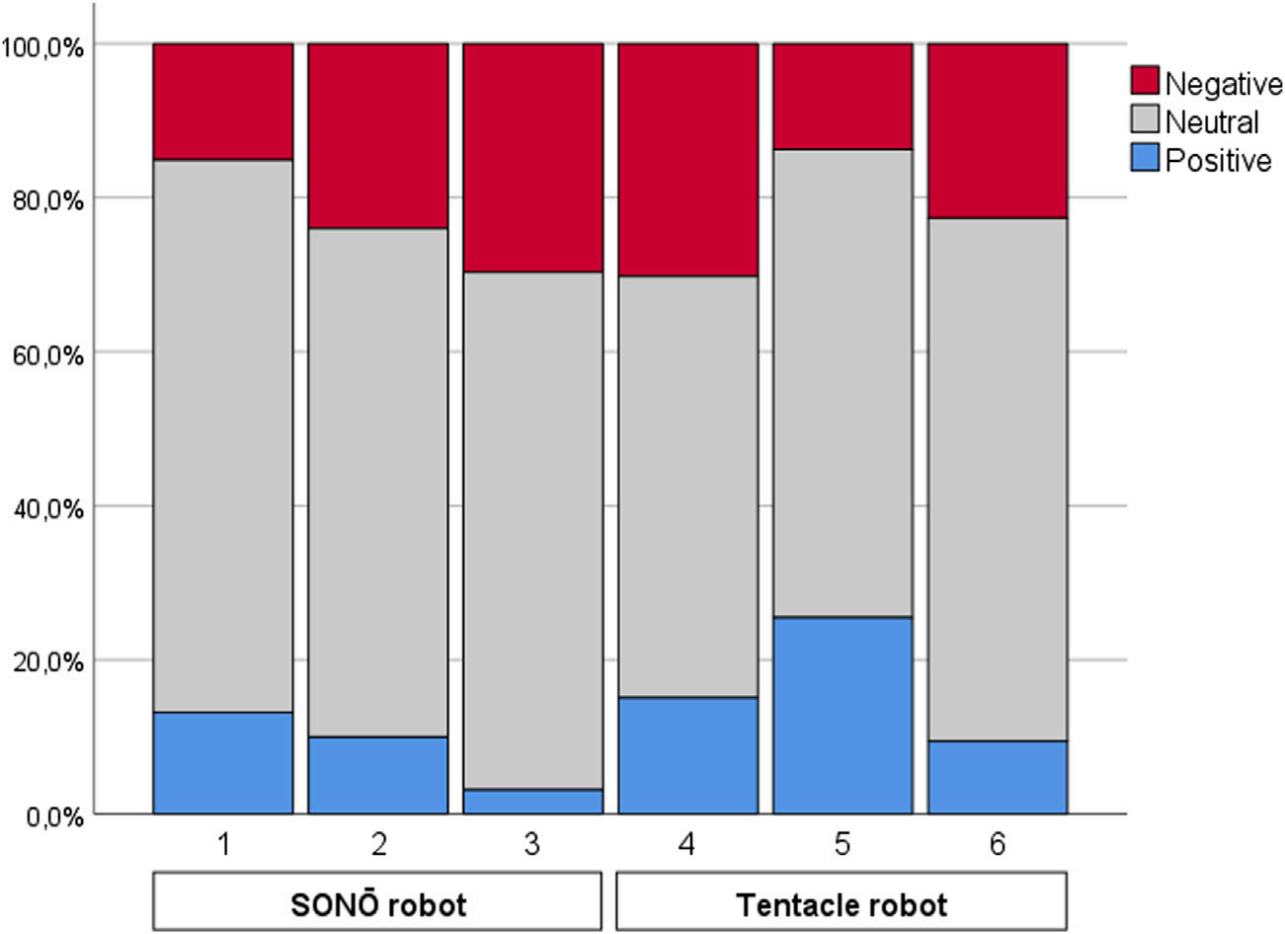
Stacked bar graph showing proportional distributions of positive, neutral, and negative sentiment words.

**FIGURE 7:**
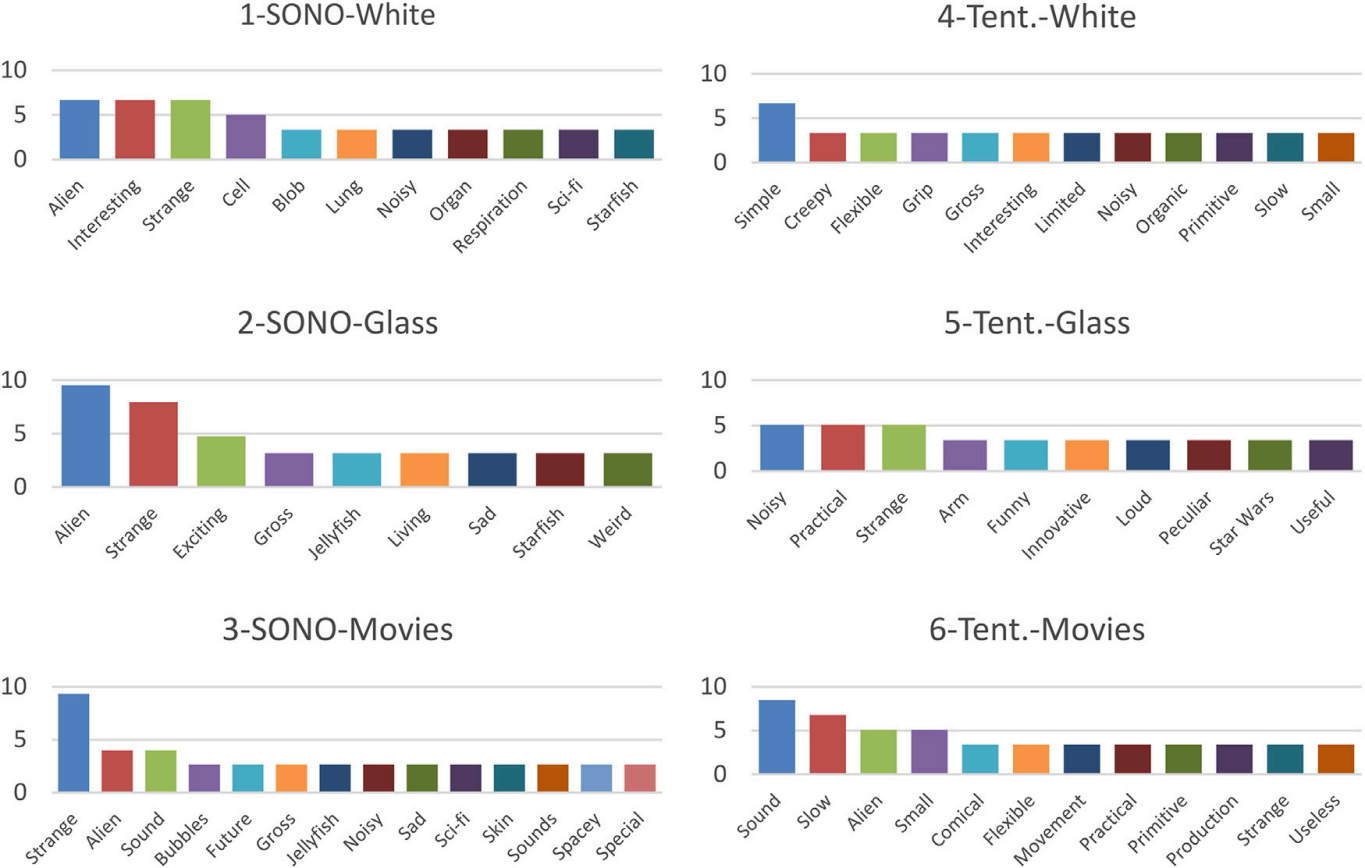
Most frequent words used by participants. The *y*-axis gives percentage of word occurrence frequency (%).

A total of 378 responses were obtained, corresponding to 3 word entries from each of the 126 participants. The three response items from one participant who had used the three words to form one coherent entry (“star,” “wars,” “sounds”) were reduced to two items (“star wars” and “sounds”) and a blank entry, yielding a reduction to 377 items. As the next step, all Danish items were translated into English and items containing more than one word were shortened to one word, following the procedure in (Damholdt et al., 2019). Two coders then coded all words as being of either negative, neutral, or positive sentiment. Cohen’s κ was run and yielded a substantial interrater reliability of *κ* = 0.736, the percentage of agreement was 85.9% with 324 out of 377 words categorized identically by the two coders. The identically categorized words were included for further analysis (proportional distributions for each condition are visualized in Figure 6 and most frequent words, mentioned by two or more participants, are visualized in Figure 7).

Neutral words were subsequently excluded, which yielded a total of 114 either negative or positive words that were included in the logistic regression. Direct logistic regression was performed to assess the impact of a number of factors on the likelihood that a word listed by a respondent would have a positive sentiment. The model contained 5 independent variables (condition, gender, age, familiarity with robots, faculty).

For Experiment 1, the full model was statistically significant χ2 (15, *N* = 53) = 31.6, *p* = 0.007, indicating that the model was able to distinguish between words with a positive and negative sentiment. The model explained between 44.9% (Cox and Snell R square) and 65.6% (Nagelkerle R squared) of the variance in positive and negative words, and correctly classified 84.9% of cases. However, none of the independent variables made a unique statistically significant contribution to the model, with only gender (*p* = 0.054) and age (*p* = 0.066) being trend level significant.

The full model was also statistically significant χ2 (15, *N* = 61) = 31.34, *p* = 0.005, for Experiment 2, indicating that the model was able to distinguish between words with a positive and negative sentiment. The model explained between 40.2% (Cox and Snell R square) and 54.0% (Nagelkerle R squared) of the variance in positive and negative words, and correctly classified 82.0% of cases. However, none of the independent variables made a unique statistically significant contribution to the model.

### Thematic Analysis

In the questionnaire, participants were asked to indicate to which extent they agreed with the following statement: “The robot has a sound that is appropriate for it.” To gain further insights into people’s perceptions of the different sound designs, we also asked people to elaborate on their chosen answer. We analyzed the replies by *thematic analysis*, using an inductive coding to allow for unexpected themes to emerge (Braun and Clarke, 2006).

Two coders read through all replies obtained in both experiments and respectively identified 6 and 8 codes for recurrent utterances. The coders shared their codes with each other (of which 5 were overlapping) and merged them into a coding scheme with 9 codes capable of adequately capturing and differentiating salient participant utterances. We chose to include all codes because of the thematic analysis having an exploratory aim. A codebook (included as Supplementary Material) was created and both coders coded the data using this coding scheme. Data items (individual participant responses) were assigned from 1 to 3 matching codes each and then exported into separate lists for each code. Finally, the first coder inductively constructed 6 recurrent themes from the lists of items, which we describe below and illustrate with exemplary quotes.

#### Theme #1: Loud Sound

15 participants mentioned the sound’s loudness or described it as shrilling or noisy. Some (*N* = 8) explicitly expressed positive or negative opinions about the sounds. The positive comments (*N* = 2) focused on how the sound was loud but suited the robot and did not cause irritation:

“*The sound is a bit loud, but not noisy or unpleasant*” (Participant 94, 5-Tent.-Glass)

The negative comments (*N* = 6) expressed annoyance, stress, and discomfort upon experiencing the sound:

*“The sound was disturbing and a bit too loud, which I felt did not suit the robot”* (Participant 39, 2-SONŌ-Glass).

The majority (*N* = 9) of the 15 items in the theme come from “Glass Attack” sound design conditions [2-SONŌ-Glass (*N* = 4); 5-Tent.-Glass (*N* = 5)]. 8 of these describe this sound design as being too loud or annoying.

#### Theme #2: Othering Robot/Sound

Nearly a quarter of all participants (*N* = 30) described the robot and/or its sound through what we refer to as “othering.” By this term we describe utterances that position the robot or its sound as something that differs from or falls outside of what is deemed to be normal and relatable. This encompasses descriptions of it as either 1) strange, weird, or mystical or 2) alien-like, science fiction (sci-fi)-like, otherworldly, or futuristic. While there were 20 instances of this for the SONŌ robot [1-SONŌ-White (*N* = 5); 2-SONŌ-Glass (*N* = 8); 3-SONŌ-Movies (*N* = 7)], there were only 10 for the Tentacle robot [4-Tent.-White (*N* = 2); 5-Tent.-Glass (*N* = 3); 6-Tent.-Movies (*N* = 5)].

12 participants described the robot or its sound as strange, peculiar, mystical, or weird and most (*N* = 7) commented negatively on this:

*“The sound is strange and so is the robot. But a more pleasant sound could be more suiting”* (Participant 23, 2-SONŌ-Glass).

*“I found it strange, maybe unnecessary, to add an artificial sound to the actions”* (Participant 90, 5-Tent.-Glass).

Only 2 participants described the “White Noise” sound design as strange [1-SONŌ-White (*N* = 2)]. The “Glass Attack” and “Movies” sound designs, on the other hand, were described as such 6 and 4 times respectively [2-SONŌ-Glass (*N* = 5); 5-Tent.-Glass (*N* = 1); 3-SONŌ-Movies (*N* = 3); 6-Tent.-Movies (*N* = 1)]. Descriptions that refer to the sound as strange principally came from Experiment 1 that used the SONŌ robot (*N* = 10), and of these, 4 additionally described the robot’s appearance as strange.

18 participants instead found the robot or its sounds to be otherworldly, futuristic, or conjure up aliens or sci-fi. Some found the sound “too futuristic” (participant 99, 5-Tent.-Glass) or “overly scifi (sic) sounding” (participant 112, 6-Tent.-Movies) or stated that:

*“The sound is okay, perhaps a bit UFO like”* (Participant 124, 6-Tent.-Movies)

Others referenced specific examples from popular culture including the *Alien* movies. A third group pointed out that it was only some qualities of the sound that made it appear “alien” whereas other qualities contributed to the experience of the robot as being a living, autonomous, or even sentient creature.

Comments relating to sci-fi, aliens, and the future predominantly concern the “Movies” sound design [3-SONŌ-Movies (*N* = 4); 6-Tent.-Movies (*N* = 4)]. Hence, it appears that some participants were able to trace back the connection to this sound design’s original sources of inspiration.

#### Theme #3: Associations to Other Sounds

22 participants associated the sound designs with sounds emitted from familiar man-made objects, living creatures, or natural phenomena.

Such responses for the “White Noise” sound design largely concern sounds of wind, breath, or air [1-SONŌ-White (*N* = 6); 4-Tent.-White (*N* = 4)]. One participant associated 2-SONŌ-Glass with the sound of breathing, but no other associations to wind, breath, or air were present for the “Glass Attack” and “Movies” sound designs.

Interestingly, in conditions with the “White Noise” sound design, associations differ markedly for the SONŌ and the Tentacle robot. For SONŌ the sound reminded participants of the robot breathing in sync with the robot’s movements (*N* = 3). Or participants on the contrary stated that the sound of wind was not appropriate for the robot and that it instead should have had more “breathing sounds” (*N* = 3):

“*It has a breathing sound*” (Participant 4, 1-SONŌ-White)

“*My first thought was not the sound of ?wind? when I saw it”* (Participant 6, 1-SONŌ-White).

For the Tentacle robot, 1 participant argued that the “blowing sound” made the robot “more repulsive” (Participant 79, 4-Tent.-White), while another participant believed the sound to be the actual sound of the pressurized air actuating the robot and not a designed sound. Two other participants connected the sound to hydraulics, vacuum, and air-controlled machinery:

“*Because I was thinking of vacuum and the sound seems hydraulic, I find it well-suiting*” (Participant 75, 4-Tent.-White)

Some participants instead described the sound as “robot-like” (*N* = 7), mainly in the 3-SONŌ-Movies condition (*N* = 4). However, 2 participants in 5-Tent.-Glass also argued that this sound is how one would imagine a robot to sound like, and 1 participant in 6-Tent.-Movies believed that the robot “(*…*) *does not say words like humans and therefore it sounds like a robot*” (Participant 116), 6-Tent.-Movies).

While most participants did not elaborate on why or how the sound was robot-like or what a “typical” robot sounds like, 1 participant did mention specific movie examples:

*“It sounds like what one always imagines a robot to sound like. Very mechanical and a sound one has heard in Star wars/terminator [sic]”* (Participant 82, 5-Tent.-Glass).

Remaining answers within the theme (*N* = 5) associated the sound with various living creatures or objects. One participant experiencing the SONŌ robot, for instance, argued that the “(*…*) *sound was stressful, sounded like a whale*” (Participant 37, 2-SONŌ-Glass). Four participants experiencing the Tentacle robot instead associated its sound with technical equipment including an airplane (Participant 87, 5-Tent.-Glass), a car (Participant 126, 5-Tent.-Glass), a robotic arm in a factory (Participant 105, 6-Tent.-Movies), and medical equipment (Participant 77, 4-Tent.-White).

#### Theme #4: “Organic” Appearance vs. “Mechanical” Sound

14 participants answered by evaluating the connection between the robot’s visual appearance and its sound, with a majority invoking a dichotomy between organic and mechanical qualities.

For the SONŌ robot, comments (*N* = 10) predominantly described the sound as more “mechanical” or “electronic” than the “natural” or “organic” appearance of the robot (*N* = 7), and were distributed nearly evenly among the three sound designs—1-SONŌ-White (*N* = 3), 2-SONŌ-Glass (*N* = 2), and 3-SONŌ-Movies (*N* = 2):

“*It inflated with a sound that sounded more mechanical than what I would expect from something organic*” (Participant 5, 1-SONŌ-White)

“*It sounds more electronic than the organic feeling it emanates*” (Participant 59, 3-SONŌ-Movies)

Only 1 of the 4 comments for the Tentacle robot invoked a distinction between “organic” and “mechanical” (in condition 4-Tent.-White). But 7 participants commented on this for the SONŌ robot irrespective of sound design, which indicates that the SONŌ robot’s embodiment or its framing as a social robot may have contributed to participants hearing the sound as mechanical.

#### Theme #5: Does Sound Match Appearance?

15 participants evaluated the fit between the robot’s appearance and its sound for the SONŌ robot [1-SONŌ-White (*N* = 5); 2-SONŌ-Glass (*N* = 5); 3-SONŌ-Movies (*N* = 5)].

A majority of participants (*N* = 9) stated that the sound suits the robot’s looks or matches what is expected for the robot’s appearance, with “Glass Attack” having the highest prevalence [1-SONŌ-White (*N* = 2); 2-SONŌ-Glass (*N* = 4); 3-SONŌ-Movies (*N* = 3)]. Others (N = 6) argued that the sound design was surprising or inappropriate for the robot’s appearance [1-SONŌ-White (*N* = 3); 2-SONŌ-Glass (*N* = 1); 3-SONŌ-Movies (*N* = 2)]:

*“The sound’s accentuated (sic) is a bit surprising compared with the robot’s appearance”* (Participant 62, 3-SONŌ-Movies).

*“I think the sound correlates well with its appearance, sort of innocent and a bit sad”* (Participant 22, 2-SONŌ-Glass).

#### Theme #6: Synchronized Movement and Sound

For the Tentacle robot, instead of appearance, we found a focus on the connection between movement and sound in responses. Where 6 participants commented on this connection for the SONŌ robot [1-SONŌ-White (*N* = 2); 2-SONŌ-Glass (*N* = 1); 3-SONŌ-Movies (*N* = 3)], nearly twice as many (*N* = 11) did so for the Tentacle robot [4-Tent.-White (*N* = 5); 5-Tent.-Glass (*N* = 3); 6-Tent.-Movies (*N* = 3)]. 10 of these found the sound to accompany the movements well or to be e.g. “(*…*) *in harmony with the movements*” (participant 120, 6-Tent.-Movies). The remaining participant, by contrast, described the sound as erratic, unnecessary, and not matching the robot’s movements (participant 70, 4-Tent.-White). For the SONŌ robot, all 6 participants evaluated the sound designs as fitting the robot’s movement:

“*There was an adequate synchronization between the movement and the sound*” (Participant 7, 1-SONŌ-White)

*“It (the sound) accompanied the movements well, and it gave a sense of reliability to the machine”* (Participant 97, 5-Tent.-Glass).

A possible explanation for why appearance is in focus for the SONŌ robot and movement in focus for the Tentacle robot could be that the latter has more visible movement. The tentacle changes position and bends in three dimensions, whereas the SONŌ robot’s surface only inflates somewhat upward, which might to some participants not be sufficient to be regarded as “movement” and is therefore categorized as a change in the robot’s appearance instead.

## Discussion

In this article we have explored the potentials of augmenting soft robotics with sound for human-robot interaction through the design of the SONŌ robot and its associated sound designs, and presented a system to generate sound to accompany the movements of a pneumatically actuated soft robot. Our approach was based in creative practice and artistic research methodologies combined with empirical HRI methods for testing.

Surprisingly, the quantitative results from the user study indicate that we must reject our three hypotheses; the two “soft” sound designs did not lead to higher *warmth* and *competence* ratings and lower *discomfort* ratings than the third “hard” sound design. Neither were the “soft” sounds deemed more appropriate for the two soft robots and they did not elicit a higher rate of words with positive sentiment to describe the robots.

Comparing results from both experiments, we found that there was no difference in how appropriate the three sounds designs were rated to be when comparing between the two robots, i.e. none of the sound designs were deemed a better fit for one or the other of the two robots. Hence, from this result we cannot conclude that one of the designs is especially fit for a communicative soft social robot, such as SONŌ, or a soft cobot, such as the Tentacle robot.

In exploratory analyses, however, we found that in Experiment 1, the “Movies” sound design made the SONŌ robot appear significantly more *responsive* than “Glass Attack.” “Movies” also made this robot appear significantly more *aggressive* than both other two sound designs. Similarly, in Experiment 2, we obtained trend level statistically significant differences for individual RoSAS subitems: for “Movies” there was a trend toward it making the Tentacle robot less *reliable* than “Glass Attack,” and more *awkward* than “White Noise.” These results indicate that the sound designs do impact people’s perception of very specific qualities of the robots, such as *responsiveness*, *aggression*, *reliability*, and *awkwardness*, but perhaps not the broad high-level main RoSAS constructs. Moreover, that these effects differed for the two experiments suggests that sound interacts with context or morphology in determining how specific aspects of a soft robot’s sociality are evaluated.

In logistic analyses of word sentiment, we found some unexpected effects, not related to the sound designs. In Experiment 1, gender (*p* = 0.054) and age (*p* = 0.066) had marginal effects on whether a participant used a word with a positive sentiment to describe the robot. Male participants appear less likely to use a word with positive sentiment and the direction of the latter relationship matches the results obtained by Damholdt et al. (2019), where an increase in age led to a higher probability that participants would use a word with positive sentiment. This marginal effect might become significant with a wider age range, and not all but one participant being university students, as in our cohort. These effects could be interesting to study further with respect to how they compare with other effects of age on perception of and attitudes toward robots. A possible explanation for this result could be the so-called positivity effect, which describes a shift from a negativity bias in young people to a preference for positive information later in life (Carstensen and DeLiema, 2018). From Figure 6, which shows the proportional distributions of positive/neutral/negative words, we can equally observe a trend toward different ratios of positive-negative words in the different conditions, which might become significant with more statistical power.

In Figure 7, which shows bar graphs of the most used words, it can be seen that more nouns feature as recurrent descriptive words for the SONŌ robot than for the Tentacle robot, which has mostly evaluative adjectives (however, “alien” could be counted as both a verb and noun). This matches well with that the Tentacle robot was framed as a robot made for a specific practical purpose, which it is evaluated for, whereas the SONŌ robot was presented in a more open-ended scenario as a socially communicative robot.

Comparing word use between the three conditions for each robot more closely, we can see that for the SONŌ robot, the words “alien” and “strange” are both among the top three words mentioned in all conditions. Therefore, it is likely that these descriptions are independent of the three sound designs. This also matches that descriptions of this robot itself as “strange” or “alien”, are prevalent in Theme 2 of the thematic analysis. Moreover, on the RoSAS scale, “strange” is rated to have a higher association with the SONŌ robot (M:5.95-6.00) than the Tentacle robot (M:4.15-5.00), and using T-tests we could verify that these differences in mean values between the robots were significant in both the “White” and “Glass” condition (*p* = 0.002, *p* = 0.032) and close to significant for “Movies” (*p* = 0.075). Based on this we conclude that it is likely that the SONŌ robot’s embodiment or contextual framing creates the impression of the robot as being “strange” and not differences between the sound designs.

When looking at words that are used for individual sound designs for both robots, similarities are also apparent. For both conditions that used the “Movies” sound design, the word “sound” is among the two most frequently mentioned words (and the word “sounds” is additionally present for one condition). This suggests that the “Movies” sound design draws more attention to itself, than the other sound designs, which are less obtrusive and perhaps easier to integrate into the overall impression of the robot. However, either the word “noisy” or “sound” was mentioned by two or more participants in all conditions except two. That the sound of “Movies” is perceived as more dominating or assertive, aligns well with that this sound design contributed to the robot appearing more aggressive in Experiment 1.

In the thematic analysis, we uncovered 6 recurrent themes, and found differences within these in how the three sound designs were assessed qualitatively. For instance, it was predominantly the “Glass Attack” sound design that was mentioned as being loud, which the majority of participants experienced negatively (and “Noisy” and “Loud” were also among the most frequently used words to describe the Tentacle robot when it is used “Glass Attack”). Perhaps more interestingly, the thematic analysis equally showed that the three sound designs were described differently when they were used by each of the two robots. A main takeaway from the thematic analysis is thus that robot type, i.e. the robot’s embodiment combined with its specified use context, appears to affect how a sound design is understood and how the specific sounds made by a soft robot are interpreted. For instance, the associations to other well-known sounds were different for the “White Noise” sound design for the two robots: While it was associated to air for both robots, for the SONŌ robot it was associated with breathing and live organisms, whereas for the Tentacle robot it was instead pneumatics and technical equipment that was mentioned. This observed difference aligns with prior work showing that embodiment affects emotional response to nonlinguistic utterances (Wolfe et al., 2020). Yet, our study design does not allow us to determine if this difference is an effect of embodiment or of context. Further work is needed to separate and distinguish between the effects of each of the two.

Returning to the research questions posed at the outset of our inquiry, “What effect does ‘soft’ sound have on people’s social perception of a soft robot?” and “Are ‘soft’ sounds a more appropriate match for a soft embodiment?”, the conclusion to draw is that these questions need to be asked with more nuance. Differences in sound design we authors, as creative practitioners active in the fields of electronic music and robotic art, picked up on and deemed to have a marked effect on our own perception of the soft robots, might not have enough impact on people in general, so as to make a difference with respect to how they rate impressions of high-level constructs such as *warmth*, *competence*, and *discomfort*. As we have explored through our practice-based artistic research, different kinds of “soft” sound exist and as the empirical tests showed, there were qualitative differences in how the robots were perceived when utilizing the three different sound designs. In further work, it would be relevant to study, that if different sound designs do not have marked effects on a soft robot’s general sociality, then could sound perhaps affect other more basic perceived qualities of the robot? Studies have shown, for instance, that humans and animals are able to infer what material an object is made from by using visual information and impact sounds, i.e. the sound an object makes when being struck by e.g. a hammer, and that there are strong audio-visual interactions in material-category perception (Fujisaki et al., 2014). In one study, the appearance of glass combined with the impact sound of a bell pepper was thus perceived as transparent plastic (Fujisaki et al., 2014). This phenomenon is worthy of further study in relation to soft robotics, with a view to determining if sound could alter people’s perceptions of a soft robot’s affordances or stiffness, for instance. In a previous study (Jørgensen et al., 2021) we found that in interactions with humans, soft robots are sometimes spontaneously subjected to a more forceful handling than traditional robots, sometimes even to the point of them breaking. A possible way to prevent this, could be to add a sound to the robot that makes it appear softer or more fragile, and this way nudge the user to handle it more carefully.

As further work, we plan to develop the SONŌ robot and the system into a finished artwork and to conduct further user tests during its exhibition. This will allow us to gauge if the change of setting from a university classroom to an art exhibition contributes to different sound designs having more impact on people’s perceptions of the robot, e.g. due to a heightened aesthetic awareness induced by the latter context.

## Limitations

Despite offering design advantages in terms of variation, flexibility, and adaptability, FM synthesis might not be the most appropriate technique to generate “soft” sounds. Perhaps the sounds that can be created with FM synthesis are not “soft” enough, and the differences between the three sound designs are not pronounced enough to have significant effects. A limitation to the study is, that we, following common practice within artistic research, did not test whether the “soft” sounds were perceived as “soft” by lay users, or how lay users define “soft” sound, which could be done as further work. This limits the generalizability of the user study’s results to the two specific definitions of “soft” sound embodied in the “Movies” and “White Noise” sound designs. Under Theme 4 in the thematic analysis, for instance, we found that for the SONŌ robot participants remark on all three sound designs that they are more “mechanical” or “electronic” than what would be expected from this robot’s “organic” appearance. This could indicate that more “organic” sounds, such as e.g. recorded sounds, could be a better fit for this embodiment.

Another limitation of the user study is that the sounds which are generated by our system are synchronized with the movements being performed. Hence the sounds produced by a specific sound design change somewhat, due to varying durations between the two robots, but they do share the same characteristic overall quality. The movements of the tentacle, for instance, are based on sequences with longer inflation times; hence, the sounds emitted are also made longer with this robot.

In the thematic analysis it is evident that the “Glass Attack” sound design is perceived as loud by several participants. This could be due to that some frequencies are perceived as louder than others (Cook, 1999), and that this sound design made more use of these. To account for this, we could have asked people to rate the loudness of the sounds in a pretrial and adjusted to the perceived loudness in each condition of the experiments based on the pretrials, rather than doing this based on our own perception of the sound.

With respect to the RoSAS scale, there are several scale items that rely on interactivity, and due to the Covid-19 pandemic we were only able to display the robots to participants and the robots would not respond to them. This makes the assessments of e.g. *competence* less relevant and reliable.

A limitation could also result from the choice made to not prime participants to focus on sound. It is possible that the unfamiliar appearance of the soft robots contributed a novelty effect that trumps the effect of the sound design in the evaluations. I.e. the quaint looks of the robots might have stolen the focus from the sound and contributed to lessening the effect of differences in sound, which might have been more pronounced with a more common robot.

## Data Availability

The datasets presented in this article are available upon request. Requests to access the datasets should be directed to jonj@mmmi.sdu.dk

## Supplementary Material

Supplementary Data Sheet 1 Ableton patch files

Supplementary Data Sheet 2 Setup Guide: Arduino/Ableton Live MIDI connection

Supplementary Data Sheet 3 Arduino code

Supplementary Data Sheet 4 Technical Details on FM Synthesizer Patches

Supplementary Data Sheet 5 Code book – Thematic analysis
