# Supplementary material for: The Sounds of Softness. Designing Sound for Human-Soft Robot Interaction
Source: Front Robot AI. 2021 Oct 12;8:674121. doi: 10.3389/frobt.2021.674121 (PMC8546171; doi:10.3389/frobt.2021.674121)
Supplement: Supplementary file 1 [file DataSheet2.PDF]

Arduino / Ableton Live MIDI connection

How to setup a software bridge between Arduino and Ableton Live

Required:

- **Arduino 'MIDI' library** - this third-party library for Arduino must be included in order to use custom commands in Arduino.
  https://github.com/FortySevenEffects/arduino_midi_library/

- **Hairless MIDI** - a MIDI to serial bridge - software for connecting serial devices such as Arduino and sending as well as receiving MIDI signals and values.
  https://projectgus.github.io/hairless-midiserial/

- **LoopMIDI** - [Only required on Windows] - a program used to create virtual loopback MIDI-ports to interconnect applications on Windows-computers. (Not needed on Mac OSX as this feature is a built-in standard).
  http://www.tobias-erichsen.de/software/loopmidi.html

- **Ableton Live** - a music DAW that is able to send and receive MIDI signals. Other DAWs (or even hardware MIDI devices) could be used instead.

Guide:

1. Launch LoopMIDI and setup virtual port (program not needed on Mac OS X)
2. Establish USB connection to Arduino and launch Hairless MIDI. Choose inserted Arduino on list "Serial port" and choose the name of the virtual port you created in LoopMIDI on lists "MIDI In" and "MIDI Out". Remember to tick on box "Serial <-> MIDI Bridge On".
3. Launch Ableton Live. Make sure that the virtual MIDI port is registered under "Preferences > MIDI".
4. The connection between Arduino and Ableton should now be established and you should see (and hear if you have armed a track within Ableton) that signals from Arduino are being sent to Ableton.
5. If you wish to alter the Arduino's code, remember to deactivate connection between Arduino and Ableton in Hairless MIDI by ticking off box "Serial <-> MIDI Bridge On".
