# Supplementary material for: The Sounds of Softness. Designing Sound for Human-Soft Robot Interaction
Source: Front Robot AI. 2021 Oct 12;8:674121. doi: 10.3389/frobt.2021.674121 (PMC8546171; doi:10.3389/frobt.2021.674121)
Supplement: Supplementary file 3 [file DataSheet4.PDF]

FM Synthesizer Patches - *Ableton Live*'s "Operator"

	Algorithm	Operator settings	Filter settings	LFO settings	Pitch envelope settings	Additional effects
"White Noise"	Carrier operators A and B placed next to each other:<br>**A & B**	Operator A (-12 dB): White noise signal<br>*[1 s attack time, 600 ms decay, 816 ms release]*<br>Operator B (-20 dB): custom shaped square wave ranging between 10 - 110 Hz<br>*[20 s attack time, 600 ms decay, 291 ms release]*	Cutoff filter which ranges between 250 Hz and 10 kHz (250 when macro is at 0 -> 10 kHz when macro is at 127) and set to 20% resonance<br>*[Filter attack 0 ms at 0%, decay 20.5 s at 67 %, sustain 39 %, release 1.5 s at]*<br>Filter end position ranges between 0% at 0 and 100% at 127<br>FIlter envelope ranges between 0% at 0 and 100% at 127	LFO set to 116 (low) sine at 19% intensity<br>*[LFO attack 534 ms, 2.5 s decay, 2.11 s release]*<br>LFO destinations: Carrier operator pitch A & B, carrier operator B volume, filter cutoff	Pitch envelope set to 100%<br>*[Pitch envelope attack 44.7 ms at +12 semitones, decay 600 ms at +48 semitones, sustain at 0 semitones, release 2.38 s at -48 semitones]*<br>Pitch envelope destinations: carrier operator pitch A & B, carrier operator A volume, filter cutoff	Reverb: 2.3 s decay time, hi cut, 85% density, 0.67 size
"Glass Attack"	Modulator operator D modulates modulator operator C which modulates both carrier operator A and B:<br>**D > C > A & B**	Operator A (-5 db): sine wave ranging between 160 Hz and 460 Hz<br>*[37 ms attack time, 60 s decay, 2.29 s release]*<br>Operator B (-43 dB): sine wave ranging between 2.66 kHz and 5.66 kHz<br>*[49 ms attack time, 4.55 s decay, 5.89 s release]*<br>Operator C (-53 dB): sine wave ranging between 300 Hz and 3.3 kHz<br>*[646 ms attack time, 60 s decay, 15.2 s release]*<br>Operator D (-42 dB): sine wave ranging between 7.04 kHz and 10 kHz<br>*[9.33 s attack time, 600 ms decay, 50 ms release]*	Morph state variable filter at 132 Hz and set to 61% resonance - morph set to 18 high pass/notch<br>*[Filter attack 39.2 ms at 10%, decay 12.7 s at 100%, sustain 0%, release 4.96 s at 100%]*<br>Filter envelope set to 100%	LFO set to 127 (high) sine at 56% intensity<br>*[LFO attack 1 s, 1.77 s decay, 889 ms release]*<br>LFO destinations: Filter	- - - - - - - - - - - - -	- - - - - - - - - - - - -
"Movies"	Carrier operators A and B placed next to each other:<br>**A & B**	Operator A (-12 dB): Square wave ranging between 43.3 Hz and 252 Hz<br>*[301 ms attack time, 4.96 s decay, 447 ms release]*<br>Operator B (-22 dB): Sawtooth wave ranging between 16.1 Hz and 86.6 Hz<br>*[0 ms attack time, 2.49 s decay, 631 ms release]*	Cutoff filter ranges between 4.8 kHz and 13.7 kHz and is set to 20% resonance<br>*[Filter attack 0 ms at 0%, filter decay 25.4 s at 100%, sustain at 0%, release 1.93 s at 100%]*<br>Filter envelope is set to 0%	LFO set to 70.56 (low) sine at 25% intensity<br>*[LFO attack 154 ms, 2.96 s decay, 291 ms release]*<br>LFO destinations: Filter	- - - - - - - - - - - - -	Echo: 1ms delay, 93% feedback<br>*[Echo wobble: 100%, 50]*
