# Supplementary material for: The Sounds of Softness. Designing Sound for Human-Soft Robot Interaction
Source: Front Robot AI. 2021 Oct 12;8:674121. doi: 10.3389/frobt.2021.674121 (PMC8546171; doi:10.3389/frobt.2021.674121)
Supplement: Supplementary file 5 [file DataSheet5.PDF]

CODEBOOK

Code name	Code definition	When to use	When not to use	Example
1. Natural vs. artificial	The robot's sound is described with words that imply it is either manmade or similar to a natural organism	Use when a person mentions either of the words: mechanic, artificial, organic, natural	When a person mentions the words "alien" or "sci-fi" (Code 5).<br>When a person mentions wind/breath/air (Code2)	*" It sounded like the robot is breathing and this made it look more realistic and organic"*
2. Wind/breath/ air	The robot's sound gives associations to wind/breath/air	Use when a person compares the robot's sound to wind/breath/air		*"It is just the sound of air"*
3. Loud/noisy	Descriptions of the sound as loud or noisy	Use when a person mentions the loudness or noise level of the robot's sound		*" The sound is a bit loud, but not noisy or unpleasant"*
4. Robot-like sound	Sound from the robot reminds a person of sounds from other robots (e.g. from popular culture)	Use when a person compares the sound to their idea of a typical "robot sound" or specific real or fictional robots		*""It is a robot sound, so it's kinda what i imagine a robot or a machine would sound like"*
5. Othering	Robot or sound is described as other or "alien"	Use when a person states that the robot and/or its sound are strange/weird/ alien- or sci-fi-like	When a person describes the robot or its sounds as a natural organism or as manmade (Code 1). When a person compares the sound to a known robot from sci-fi – e.g. R2D2 (Code 4)	*" Seemed overly 'Sci-fi' sounding, and the sound didn't much indicate anything"*
6. Sentiment	Sentiments expressed towards the robot or its sound	Use when a person expresses sympathy/antipathy or positive/negative feelings towards the robot or its sound		*"The sound was disturbing and a bit too loud which I didn't think suited the robot"*
7. Relation between appearance and sound	The robot's appearance and its sound are compared	Use when a person comments on the relation between the robot's appearance and its sound	When a person only comments on the connection between the robot's sound and its movements (Code 8)	*"I think that the robot's look fits its sound "*
8. Connection between movement and sound	A connection between the movements and sounds is mentioned	Use when a person describes a connection between the movements and sound made by the robot	When a person only comments on the relation between the robot's sound and its appearance (Code 7)	*"The sound is in harmony with its [the robot's] movements"*
9. Sounds like...	The robot's sound is compared to other sounds	Use when a person compares or associates the robot's sound to specific other sounds	When a person compares the sound to the sound of wind, breathing, air (Code 2), sci-fi , alien (Code 5), or robot-like sounds (Code 4)	*"The sound was stressful, sounded like a whale"*
